# Effectiveness of systemic thrombolysis on clinical outcomes in high-risk pulmonary embolism patients with venoarterial extracorporeal membrane oxygenation: a nationwide inpatient database study

**DOI:** 10.1186/s40560-023-00651-w

**Published:** 2023-02-06

**Authors:** Yuji Nishimoto, Hiroyuki Ohbe, Hiroki Matsui, Mikio Nakajima, Yusuke Sasabuchi, Yukihito Sato, Tetsuya Watanabe, Takahisa Yamada, Masatake Fukunami, Hideo Yasunaga

**Affiliations:** 1 Division of Cardiology, Osaka General Medical Center, Osaka, Japan; 2 Department of Clinical Epidemiology and Health Economics, School of Public Health, The University of Tokyo, 7-3-1 Hongo, Bunkyo-Ku, Tokyo, 1130033 Japan; 3 Emergency Life-Saving Technique Academy of Tokyo, Foundation for Ambulance Service Development, Tokyo, Japan; 4 Data Science Center, Jichi Medical University, Tochigi, Japan; 5 Department of Cardiology, Hyogo Prefectural Amagasaki General Medical Center, Amagasaki, Japan

**Keywords:** Thrombolytic therapy, Extracorporeal membrane oxygenation, Mortality, Pulmonary embolism, Propensity score

## Abstract

**Background:**

Current guidelines recommend systemic thrombolysis as the first-line reperfusion treatment for patients with high-risk pulmonary embolism (PE) who present with cardiogenic shock but do not require venoarterial extracorporeal membrane oxygenation (VA-ECMO). However, little is known about the optimal reperfusion treatment in high-risk PE patients requiring VA-ECMO. We aimed to evaluate whether systemic thrombolysis improved high-risk PE patients’ outcomes who received VA-ECMO.

**Methods:**

This was a retrospective cohort study using the Japanese Diagnosis Procedure Combination inpatient database from July 2010 to March 2021. We identified patients who were diagnosed with PE and received VA-ECMO on the day of admission. Patients who received systemic thrombolysis with monteplase or urokinase within two days of initiating VA-ECMO were defined as the thrombolysis group and the remaining patients as the control group. The primary outcome was in-hospital mortality and secondary outcomes were favorable neurological outcomes, length of hospital stay, VA-ECMO duration, total hospitalization cost, major bleeding, and blood transfusion volume. Propensity-score inverse probability of treatment weighting (IPTW) was performed to compare the outcomes between the groups.

**Results:**

Of 1220 eligible patients, 432 (35%) received systemic thrombolysis within two days of initiating VA-ECMO. Among the unweighted cohort, patients in the thrombolysis group were less likely to have poor consciousness at admission, out-of-hospital cardiac arrest, and left heart catheterization. After IPTW, the patient characteristics were well-balanced between the two groups The crude in-hospital mortality was 52% in the thrombolysis group and 61% in the control group. After IPTW, in-hospital mortality did not differ significantly between the two groups (risk difference: − 3.0%, 95% confidence interval: − 9.6% to 3.5%). There were also no significant differences in the secondary outcomes. Sensitivity analyses showed a significant difference in major bleeding between the monteplase and control groups (risk difference: 6.9%, 95% confidence interval: 1.7% to 12.1%), excluding patients who received urokinase. There were no significant differences in the other sensitivity and subgroup analyses except for the total hospitalization cost.

**Conclusions:**

Systemic thrombolysis was not associated with reduced in-hospital mortality or increased major bleeding in the high-risk PE patients receiving VA-ECMO. However, systemic thrombolysis with monteplase was associated with increased major bleeding.

**Supplementary Information:**

The online version contains supplementary material available at 10.1186/s40560-023-00651-w.

## Background

Acute pulmonary embolism (PE) is the most serious clinical manifestation of venous thromboembolism and is associated with substantial morbidity and mortality [1, 2]. The risk of acute PE is classified into low, intermediate, and high, depending on the risk of early death based on hemodynamic instability, right ventricular dysfunction, and comorbidities [3]. High-risk PE is an immediately life-threatening situation defined by hemodynamic instability, including cardiac arrest, obstructive shock, or persistent hypotension [3]. Among these patients with a hemodynamic compromise requiring venoarterial extracorporeal membrane oxygenation (VA-ECMO), in-hospital mortality was quite high at approximately 62% [4, 5].

Current guidelines recommend systemic thrombolysis as the first-line reperfusion treatment for patients with high-risk PE [3]. However, this recommendation is mainly based on evidence regarding PE patients with cardiogenic shock not requiring VA-ECMO [6], and little is known about the optimal reperfusion treatment in high-risk PE patients requiring VA-ECMO. There are no randomized controlled trials in high-risk PE patients requiring VA-ECMO due to the nature of the patient population with life-threatening conditions. Only two previous observational studies including patients with surgical embolectomy partially assessed the effect of systemic thrombolysis in combination with VA-ECMO on in-hospital mortality, and they showed inconsistent results [5, 7]. To our knowledge, no previous studies have focused on the effect of systemic thrombolysis in combination with VA-ECMO on in-hospital mortality.

The present study, therefore, aimed to evaluate the effect of systemic thrombolysis on in-hospital mortality and other clinical outcomes including the neurologic outcomes, hospitalization cost, and bleeding events in high-risk PE patients who received VA-ECMO, using a nationwide inpatient database in Japan.

## Methods

### Design and ethical statement

This was a retrospective cohort study using an inpatient administrative database, and the study conformed to the RECORD statement reporting guidelines [8]. This study was conducted in accordance with the amended Declaration of Helsinki and was approved by the Institutional Review Board of The University of Tokyo (approval number, 3501-(5); 19 May 2021). Because the data were anonymous, the Institutional Review Board waived the requirement for informed consent. No information about individual patients, hospitals, or treating physicians was available.

### Data source

We used the Japanese Diagnosis Procedure Combination inpatient database, which contains administrative claims data and discharge abstracts from more than 1500 acute care hospitals and covers approximately 90% of all tertiary emergency hospitals in Japan [9]. The database includes the following patient-level data for all hospitalizations: age, sex, diagnoses (main diagnosis, admission-precipitating diagnosis, most resource-consuming diagnosis, second-most resource-consuming diagnosis, comorbidities present on admission, and complications arising after admission) recorded with the *International Classification of Diseases, 10th Revision (ICD-10)* codes, daily procedures recorded using Japanese medical procedure codes, daily drug administration, and discharge status [9]. A previous validation study showed the specificity of the recorded diagnoses in the database exceeded 96%, the sensitivity of the diagnoses ranged from 50 to 80%, and the specificity and sensitivity of procedures both exceeded 90% [24].

### Study population

Using the Japanese Diagnosis Procedure Combination inpatient database from July 2010 to March 2021, which was the maximum period available at that time, we identified hospitalized patients with the primary diagnosis of PE (ICD-10 code: I26) and who received VA-ECMO on the day of admission. We did not include patients with a suspected diagnosis of PE and patients who developed PE as a complication after hospitalization. We excluded patients who received surgical embolectomy without systemic thrombolysis within two days of admission.

### Group assignment

Patients who received systemic thrombolysis within two days of initiating VA-ECMO were defined as the thrombolysis group because the current guidelines suggest that systemic thrombolysis is most effective when initiated within 48 h of the symptom onset [3], and the remaining patients were defined as the control group. Patients who received monteplase or urokinase were defined as receiving systemic thrombolysis.

### Covariates and outcomes

Covariates included age, sex, smoking history, body mass index at admission, Japan Coma Scale (JCS) at admission [10], out-of-hospital cardiac arrest (OHCA), comorbidities (coronary artery disease, heart failure, chronic lung disease, hypertension, diabetes mellitus, chronic kidney disease, or cancer), ambulance use, weekend admission, intensive care unit admission, high care unit admission, procedures on the day of admission (cardiopulmonary resuscitation [CPR], defibrillation, pacing, targeted temperature management besides VA-ECMO, right heart catheterization, or left heart catheterization), and resuscitative drugs on the day of admission (adrenaline [epinephrine], vasopressin, atropine, or amiodarone).

The primary outcome was in-hospital mortality. The secondary outcomes were favorable neurological outcomes, length of hospital stay, VA-ECMO duration, total hospitalization cost, major bleeding, and blood transfusion volume. The favorable neurological outcomes were defined as patients with a JCS of 0 (alert) or 1–3 (dizziness) at discharge. A score of 0 or 1–3 under the JCS is roughly synonymous with a Cerebral Performance Category (CPC) score of 1 or 2 [11, 12]. The detailed JCS scoring and conversion methods from the JCS to the GCS are shown in Additional file 1: Table S1 [13]. Major bleeding was defined as the presence of intracranial bleeding (ICD-10 code: I61), intraspinal bleeding (G951), pericardial hematoma (I312), intra-abdominal or retroperitoneal hematoma (K661), intra-articular bleeding (M250), intraocular bleeding (H448), and/or compartment syndrome (M622), which was in accordance with the International Society of Thrombosis and Haemostasis definitions [14].

### Statistical analysis

We used the inverse probability of treatment weighting (IPTW) by propensity scores to compare the outcomes between the thrombolysis and control groups [15, 16]. We applied a multivariable logistic regression model to predict the propensity scores for patients receiving systemic thrombolysis within two days of initiating VA-ECMO, using all the variables listed in Table 1 as predictor variables. We used the stabilized average treatment effect weight, which allowed us to maintain the total sample size of the original data and provided a conservative interval estimate of the variance of the main effect and controls for a type I error as compared to the non-stabilized IPTW [17]. We calculated the absolute standardized differences of each covariate in the unweighted and weighted cohorts to confirm the balance of the distribution of the covariates between the thrombolysis and control groups. When the absolute standardized differences between the two groups were less than 10%, we considered the imbalance in the distribution of the covariates to be negligible [18]. We used a weighted generalized linear model to compare the outcomes, with cluster-robust standard errors and treating individual hospitals as clusters. We calculated the risk differences and their 95% confidence intervals for outcomes using the identity link function in a weighted generalized linear model.

**Table 1:** Patient characteristics of the unweighted and weighted cohorts

	Unweighted cohort			Weighted cohort		
	Thrombolysis	Control		Thrombolysis	Control	
	(*n* = 432)	(*n* = 788)	ASD	(*n* = 433)	(*n* = 787)	ASD
Age, years, mean (SD)	58 (16)	60 (16)	10	60	59	2
Men, *n* (%)	179 (41)	311 (39)	4	40	40	1
Smoking history, *n* (%)						
Non-smoker	270 (63)	448 (57)	12	60	59	1
Current or past smoker	74 (17)	121 (15)	5	15	16	2
Unknown	88 (20)	219 (28)	17	25	25	1
Body mass index on the day of admission, kg/m^2^, *n* (%)						
< 18.5	12 (3)	37 (5)	10	5	4	3
18.5–24.9	175 (41)	300 (38)	5	40	40	1
25.0–29.9	111 (26)	193 (24)	3	25	25	0
≥ 30.0	45 (10)	72 (9)	4	9	10	2
Missing	89 (21)	186 (24)	7	22	22	1
Japan Coma Scale on the day of admission, *n* (%)						
0 (alert)	127 (29)	159 (20)	22	23	23	1
1–3 (dizzy)	50 (12)	59 (7)	14	9	9	0
10–30 (somnolent)	21 (5)	50 (6)	7	6	6	3
100–300 (coma)	234 (54)	520 (66)	24	62	62	1
Out-of-hospital cardiac arrest, *n* (%)	206 (48)	485 (62)	28	57	57	1
Comorbidities, *n* (%)						
Coronary artery disease	22 (5)	48 (6)	4	5	6	2
Heart failure	59 (14)	100 (13)	3	13	13	0
Chronic lung disease	8 (2)	11 (1)	4	2	1	0
Hypertension	57 (13)	91 (12)	5	12	12	1
Diabetes mellitus	30 (7)	56 (7)	1	8	7	2
Chronic kidney disease	6 (1)	12 (2)	1	1	1	1
Cancer	11 (3)	45 (6)	16	4	5	4
Ambulance use, *n* (%)	385 (89)	710 (90)	3	90	90	1
Weekend admission, *n* (%)	114 (26)	176 (22)	9	25	24	2
ICU admission, *n* (%)	343 (79)	614 (78)	4	78	78	2
HCU admission, *n* (%)	69 (16)	146 (19)	7	18	18	1
Procedures on the day of admission, *n* (%)						
Cardiopulmonary resuscitation	268 (62)	468 (59)	5	61	60	1
Defibrillation	36 (8)	85 (11)	8	10	10	2
Pacing	17 (4)	33 (4)	1	4	4	1
TTM besides VA-ECMO	78 (18)	156 (20)	4	20	19	2
Right heart catheterization	59 (14)	101 (13)	3	13	13	1
Left heart catheterization	58 (13)	165 (21)	20	18	18	0
Resuscitative drugs on the day of admission						
Adrenaline, mg, mean (SD)	5 (5)	5 (6)	12	5	5	1
Vasopressin, *n* (%)	27 (6)	62 (8)	6	8	8	4
Atropine, *n* (%)	40 (9)	60 (8)	6	8	8	1
Amiodarone, *n* (%)	18 (4)	42 (5)	6	5	5	2

*ASD* absolute standardized difference, *HCU* high dependency care unit, *ICU* intensive care unit, *SD* standard deviation, *TTM* targeted temperature management, *VA-ECMO* venoarterial extracorporeal membrane oxygenation

Continuous variables were presented as the mean and standard deviation (SD), and categorical variables were presented as the number and percentage. We considered all reported p-values as two-sided and a *p* < 0.05 as statistically significant. All analyses were performed using STATA/SE 17.0 software (StataCorp).

### Subgroup analyses

We assumed that extracorporeal CPR and OHCA had a substantial impact on in-hospital mortality. Therefore, we tested the potential for effect modification of systemic thrombolysis in combination with VA-ECMO on in-hospital mortality as well as the secondary outcomes, depending on whether the patients received CPR on the day of admission or had OHCA. We performed these subgroup analyses among the weighted cohort created in the main analysis.

### Sensitivity analyses

We performed two sensitivity analyses. First, monteplase is a third-generation thrombolytic agent with a longer half-life, higher thrombus sensitivity, and more rapid lysis [19]. Therefore, we performed sensitivity analyses excluding the patients in the thrombolysis group who received urokinase within two days of initiating VA-ECMO. Second, some patients were likely to have received systemic thrombolysis after the third day of initiating VA-ECMO but were assigned to the control group. Hence, we performed sensitivity analyses excluding those patients in the control group who received systemic thrombolysis after the third day of initiating VA-ECMO. For each sensitivity analysis, we repeated the propensity-score IPTW in the same manner as for the main analysis.

## Results

**Fig. 1:**
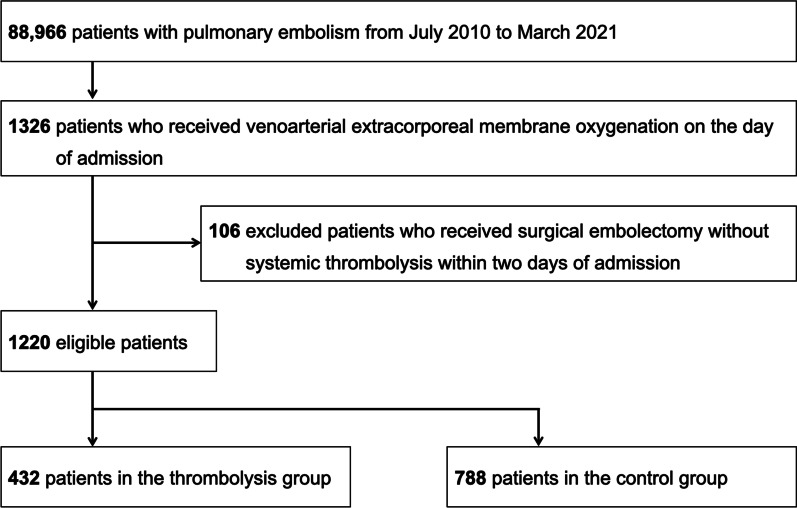
Patient flowchart

During the study period, we identified 1220 eligible patients (Fig. 1). Of those, 432 (35%) received systemic thrombolysis within two days of initiating VA-ECMO and were allocated to the thrombolysis group. Of the 432 patients in the thrombolysis group, 287 received monteplase, 117 urokinase, and 28 both monteplase and urokinase within two days of initiating VA-ECMO. Of the 788 patients in the control group, 40 received systemic thrombolysis after the third day of initiating VA-ECMO.

**Fig. 2:**
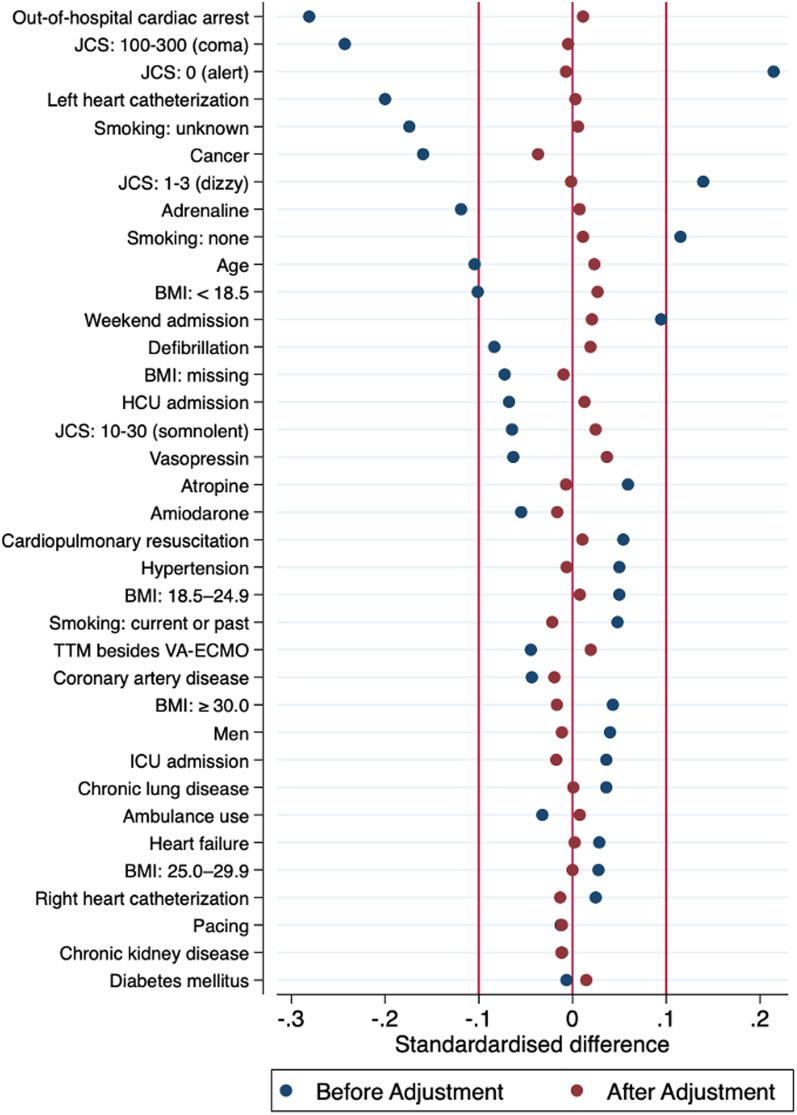
Standardized differences for each covariate before and after inverse probability treatment weighing. Red lines indicate the desired balance level. *BMI* body mass index, *HCU* high dependency care unit, *ICU* intensive care unit, *JCS* Japan Coma Scale, *TTM* targeted temperature management, *VA-ECMO* venoarterial extracorporeal membrane oxygenation

Table 1 shows the patient characteristics. Among the unweighted cohort, patients in the thrombolysis group were less likely to have poor consciousness at admission, OHCA, and left heart catheterization. After IPTW, the patient characteristics were well-balanced between the two groups (Fig. 2). The propensity score distribution between the two groups was adequately balanced after IPTW (Additional file 1: Figs. S1, S2).

Table 2 shows the outcomes in the unweighted and weighted cohorts. The crude in-hospital mortality was 52% in the thrombolysis group and 61% in the control group. After IPTW, there was no significant difference in in-hospital mortality between the two groups (risk difference: − 3.0%, 95% confidence interval: − 9.6% to 3.5%). There were also no significant differences in the secondary outcomes including the favorable neurological outcomes, length of hospital stay, VA-ECMO duration, total hospitalization cost, major bleeding, and blood transfusion volume.

**Table 2:** Results of inverse probability in the unweighted and weighted cohort

	Unweighted cohort		Weighted cohort			
	Thrombolysis	Control	Thrombolysis	Control	Risk differences	
Outcomes	(*n* = 432)	(*n* = 788)	(*n* = 433)	(*n* = 787)	(95% CI)	P-value
Primary outcome						
In-hospital mortality, *n* (%)	225 (52)	477 (61)	240 (55)	459 (58)	− 3.0 (− 9.6 to 3.5)	0.36
Secondary outcomes						
Favorable neurological outcomes, *n* (%)	182 (42)	261 (33)	170 (39)	277 (35)	4.0 (− 2.3 to 10.3)	0.21
Length of hospital stay, days, mean (SD)	29 (70)	23 (32)	27 (63)	23 (32)	3.9 (− 2.0 to 9.8)	0.20
Length of VA-ECMO, days, mean (SD)	3 (3)	3 (4)	3 (3)	3 (4)	− 0.3 (− 0.7 to 0.2)	0.21
Total hospitalization cost, × 10^3^ dollars, mean (SD)	30 (26)	27 (21)	30 (26)	27 (21)	2.9 (− 0.2 to 6.0)	0.07
Major bleeding in a critical area or organ, *n* (%)	17 (4)	22 (3)	23 (5)	21 (3)	2.6 (− 0.2 to 5.5)	0.07
Intracranial bleeding, *n* (%)	6 (1)	9 (1)	10 (2)	9 (1)	1.3 (− 0.9 to 3.5)	0.26
Blood transfusions, ml, mean (SD)						
Red blood cells	2829 (2651)	2580 (2733)	2880 (2761)	2637 (2803)	244 (− 140 to 627)	0.21
Fresh-frozen plasma	1413 (2155)	1395 (1977)	1545 (2417)	1410 (2017)	136 (− 207 to 479)	0.44
Platelet concentrate	254 (521)	290 (540)	272 (588)	297 (553)	− 26 (− 107 to 56)	0.54

*CI* confidence interval, *SD* standard deviation, *VA-ECMO* venoarterial extracorporeal membrane oxygenation

Table 3 and Additional file 1: Table S2 show the results of the subgroup analyses by CPR and OHCA in the weighted cohort, respectively. There were no significant differences in in-hospital mortality as well as the secondary outcomes.

**Table 3:** Results of the subgroup analyses by CPR in the weighted cohort

	With CPR				Without CPR			
	Thrombolysis	Control	Risk differences		Thrombolysis	Control	Risk differences	
Outcomes	(*n* = 263)	(*n* = 473)	(95% CI)	P-value	(*n* = 170)	(*n* = 314)	(95% CI)	P-value
Primary outcome								
In-hospital mortality, *n* (%)	172 (66)	309 (65)	0.3 (− 7.1 to 7.8)	0.93	67 (39)	150 (48)	− 8.5 (− 19.0 to 2.0)	0.11
Secondary outcomes								
Favorable neurological outcomes, *n* (%)	77 (29)	131 (28)	1.6 (− 5.5 to 8.7)	0.66	93 (55)	146 (47)	7.9 (− 2.5 to 18.4)	0.14
Length of hospital stay, days, mean (SD)	24 (43)	21 (33)	2.4 (− 3.7 to 8.4)	0.45	33 (85)	27 (31)	6.3 (− 5.2 to 17.9)	0.28
Length of VA-ECMO, days, mean (SD)	3 (3)	3 (4)	− 0.3 (− 0.8 to 0.3)	0.32	3 (3)	4 (4)	− 0.3 (− 1.0 to 0.4)	0.43
Total hospitalization cost, × 10^3^ dollars, mean (SD)	28 (23)	26 (21)	2.1 (− 1.5 to 5.7)	0.25	33 (29)	29 (21)	4.2 (− 1.2 to 9.5)	0.13
Major bleeding in a critical area or organ, *n* (%)	11 (4)	10 (2)	2.0 (− 1.3 to 5.3)	0.24	12 (7)	11 (4)	3.6 (− 1.9 to 9.2)	0.20
Intracranial bleeding, *n* (%)	4 (1)	3 (1)	0.9 (− 1.1 to 2.9)	0.37	6 (4)	6 (2)	1.9 (− 3.0 to 6.9)	0.45
Blood transfusions, ml, mean (SD)								
Red blood cells	2710 (2450)	2628 (2882)	81 (− 373 to 536)	0.73	3144 (3172)	2649 (2682)	495 (− 158 to 1148)	0.14
Fresh-frozen plasma	1361 (1611)	1463 (2161)	− 102 (− 420 to 216)	0.53	1831 (3280)	1329 (1776)	502 (− 180 to 1183)	0.15
Platelet concentrate	236 (421)	271 (503)	− 35 (− 109 to 38)	0.34	327 (777)	337 (619)	− 9.4 (− 179 to 160)	0.91

*CI* confidence interval, *CPR* cardiopulmonary resuscitation, *SD* standard deviation, *VA-ECMO* venoarterial extracorporeal membrane oxygenation

Table 4 and Additional file 1: Table S3 show the results of the sensitivity analyses. After excluding 145 patients in the thrombolysis group who received urokinase within two days of initiating VA-ECMO, there was also no significant difference in in-hospital mortality between the monteplase and control groups. However, there was a significant difference in major bleeding between the two groups (risk difference: 6.9%, 95% confidence interval: 1.7% to 12.1%) (Table 4). After excluding 40 patients in the control group who received systemic thrombolysis after the third day of initiating VA-ECMO, there were no significant differences in in-hospital mortality as well as the secondary outcomes except for the total hospitalization cost (Additional file 1: Table S3).

**Table 4:** Results of the sensitivity analyses after excluding patients who received urokinase within two days of initiating VA-ECMO

	Weighted cohort			
	Monteplase	Control	Risk differences	
Outcomes	(*n* = 289)	(*n* = 786)	(95% CI)	P-value
Primary outcome				
In-hospital mortality, *n* (%)	159 (55)	465 (59)	− 4.1 (− 11.6 to 3.4)	0.28
Secondary outcomes				
Favorable neurological outcomes, *n* (%)	117 (41)	271 (34)	6.1 (− 1.3 to 13.4)	0.11
Length of hospital stay, days, mean (SD)	24 (31)	23 (32)	0.7 (− 3.7 to 5.0)	0.77
Length of VA-ECMO, days, mean (SD)	3 (3)	3 (4)	− 0.4 (− 0.9 to 0.06)	0.09
Total hospitalization cost, × 10^3^ dollars, mean (SD)	29 (19)	27 (21)	1.6 (− 1.3 to 4.5)	0.28
Major bleeding in a critical area or organ, *n* (%)	28 (10)	21 (3)	6.9 (1.7 to 12.1)	0.01
Intracranial bleeding, *n* (%)	12 (4)	8 (1)	3.1 (− 0.9 to 7.2)	0.13
Blood transfusions, ml, mean (SD)				
Red blood cells	2903 (2722)	2635 (2821)	268 (− 179 to 715)	0.24
Fresh-frozen plasma	1505 (1733)	1414 (2030)	92 (− 196 to 380)	0.53
Platelet concentrate	265 (446)	297 (555)	− 31 (− 103 to 40)	0.39

*CI* confidence interval, *SD* standard deviation, *VA-ECMO* venoarterial extracorporeal membrane oxygenation

## Discussion

The present study showed no significant association between systemic thrombolysis and in-hospital mortality or the other clinical outcomes (neurologic outcomes, hospitalization cost, or bleeding events) in high-risk PE patients who received VA-ECMO. These findings were consistent in the subgroup analyses in the patients with and without CPR or OHCA on the day of admission. However, in the sensitivity analyses, systemic thrombolysis with monteplase was associated with increased major bleeding.

Unlike our results, a previous study using a nationwide inpatient database in Germany included 2197 high-risk PE patients with VA-ECMO and showed that thrombolysis in combination with VA-ECMO was associated with a lower risk of in-hospital mortality (odds ratio, 0.60 [95% confidence interval, 0.43–0.85]). In the previous study, only age, sex, and comorbidities were adjusted [5]. However, the present study showed that the control group was more likely to have poor consciousness or OHCA than the thrombolysis group in the unweighted cohort. That suggested that the effect of thrombolysis in the previous study could have been overestimated. Other differences included the prevalence of applying VA-ECMO in PE patients. In the previous study, VA-ECMO was applied to only 0.2% of all hospitalized patients diagnosed with PE (*n* = 2197/1,172,354), while it was applied to 1.5% of hospitalized patients with the primary diagnosis of PE (*n* = 1326/88,966) in the present study. This may be partly due to the differences in the study population, study periods (2005–2018 vs. 2010–2021), and national health insurance system [20].

VA-ECMO alone restores hemodynamics with right ventricular unloading and adequate tissue oxygenation [21]. During about four days of hemodynamic stabilization carried out by VA-ECMO, heparin-induced endogenous thrombolysis usually allows for weaning from VA-ECMO support [21, 22]. The present study showed no significant survival or other benefits from adding systemic thrombolysis in high-risk PE patients who received VA-ECMO. When the thrombolytic agent was limited to monteplase, systemic thrombolysis in combination with VA-ECMO was associated with increased major bleeding without improving survival. That suggested that it may be better to avoid using monteplase and consider other rescue reperfusion treatments, including surgical embolectomy and percutaneous catheter-directed treatment, for high-risk PE patients who received VA-ECMO but still had hemodynamic compromise or early recurrent PE.

The present study had several strengths. First, the present study was based on one of the largest databases, which covered approximately 90% of all tertiary emergency hospitals in Japan. Second, the present study focused not only on the qualitative assessment of the presence or absence of major bleeding but also on the quantitative assessment of blood transfusion volume.

The present study had several limitations. First, the decision on whether to use systemic thrombolysis was at the individual clinician’s discretion due to the nature of the present study using the observational database, which led to confounding by indication. We attempted to control for this confounding by indication using the IPTW. However, we were unable to control for any possible unmeasured variables, such as vital signs or laboratory data. Second, the present study was unable to identify whether patients received VA-ECMO after failing systemic thrombolysis or whether they initially received VA-ECMO and then systemic thrombolysis within two days of admission. Third, the incidence of major bleeding events in the present study was considerably lower than in the previous German study (intracranial bleeding, 1.2% vs. 4.9%) [5] and other previous studies [7, 23]. Given that the sensitivity of the diagnosis might have been low in our database [24], there was a possibility of underreporting in the major bleeding events. Fourth, 34% of the thrombolytic agents in the present study were urokinase, suggesting caution should be taken when applying our results to those in other countries where tissue plasminogen activator is the main thrombolytic agent [25]. Finally, using the JCS status at discharge might not be suitable to define whether there were favorable or unfavorable neurological outcomes as compared to the CPC or modified Rankin Scale, which were commonly used in previous studies [26].

## Conclusions

The present study showed that the use of systemic thrombolysis was not associated with reduced in-hospital mortality as well as increased major bleeding in high-risk PE patients who received VA-ECMO. However, systemic thrombolysis with monteplase was associated with increased major bleeding.

## Supplementary Information

**Additional file 1: Fig. S1.** Distribution of the propensity scores in the thrombolysis and control groups in the unweighted cohort. **Fig. S2**. Distribution of the propensity scores in the thrombolysis and control groups in the weighted cohort by inverse probability of treatment weighting analyses. **Table S1**. The JCS scoring and conversion methods from the JCS to the GCS. **Table S2**. Results of the subgroup analyses by OHCA in the weighted cohort. **Table S3**. Results of the sensitive analyses after excluding the patients in the control group who received systemic thrombolysis after the third day of initiating VA-ECMO.

## Data Availability

The datasets analyzed during the current study are not publicly available owing to contracts with the hospitals providing the data to the database.

## Abbreviations

CPC: Cerebral performance category
CPR: Cardiopulmonary resuscitation
ICD-10: International Classification of Diseases, 10th Revision
IPTW: Inverse probability of treatment weighting
JCS: Japan Coma Scale
OHCA: Out-of-hospital cardiac arrest
PE: Pulmonary embolism
SD: Standard deviation
VA-ECMO: Venoarterial extracorporeal membrane oxygenation
