# Supplementary material for: Effectiveness of systemic thrombolysis on clinical outcomes in high-risk pulmonary embolism patients with venoarterial extracorporeal membrane oxygenation: a nationwide inpatient database study
Source: J Intensive Care. 2023 Feb 6;11:4. doi: 10.1186/s40560-023-00651-w (PMC9901114; doi:10.1186/s40560-023-00651-w)
Supplement: Supplementary file 1 — Additional file 1: Fig. S1. Distribution of the propensity scores in the thrombolysis and control groups in the unweighted cohort. Fig. S2. Distribution of the propensity scores in the thrombolysis and control groups in the weighted cohort by inverse probability of treatment weighting analyses. Table S1. The JCS scoring and conversion methods from the JCS to the GCS. Table S2. Results of the subgroup analyses by OHCA in the weighted cohort. Table S3. Results of the sensitive analyses after excluding the patients in the control group who received systemic thrombolysis after the third day of initiating VA-ECMO. [file 40560_2023_651_MOESM1_ESM.docx]

**Supplementary Materials**

**Effectiveness of systemic thrombolysis on clinical outcomes in high-risk pulmonary embolism patients with venoarterial extracorporeal membrane oxygenation: a nationwide inpatient database study**

Yuji Nishimoto^1^; Hiroyuki Ohbe^2^; Hiroki Matsui^2^; Mikio Nakajima^2,3^; Yusuke Sasabuchi^4^; Yukihito Sato^5^; Tetsuya Watanabe^1^; Takahisa Yamada^1^; Masatake Fukunami^1^; Hideo Yasunaga^2^.

^1^Division of Cardiology, Osaka General Medical Center, Osaka, Japan. ^2^Department of Clinical Epidemiology and Health Economics, School of Public Health, The University of Tokyo, Tokyo, Japan. ^3^Emergency Life-Saving Technique Academy of Tokyo, Foundation for Ambulance Service Development, Tokyo, Japan. ^4^Data Science Center, Jichi Medical University, Tochigi, Japan. ^5^Department of Cardiology, Hyogo Prefectural Amagasaki General Medical Center, Amagasaki, Japan.

**Fig. S1.** Distribution of the propensity scores in the thrombolysis and control groups in the unweighted cohort

**
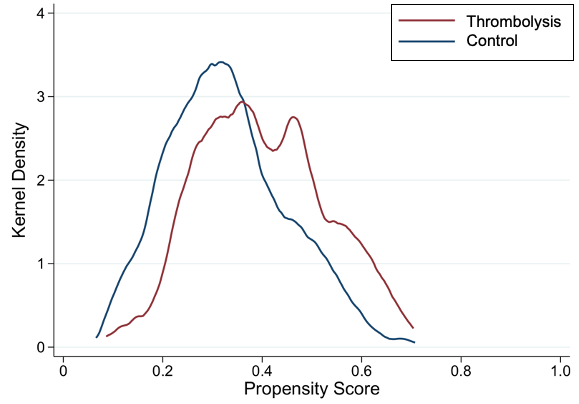
**

**Fig. S2.** Distribution of the propensity scores in the thrombolysis and control groups in the weighted cohort by inverse probability of treatment weighting analyses

**
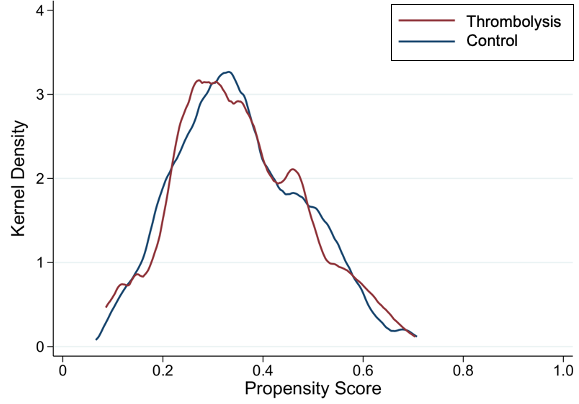
**

| **Table S1.** The JCS scoring and conversion methods from the JCS to the GCS | | |
| --- | --- | --- |
| Level of consciousness | JCS | GCS conversion |
| Alert | 0 | 15 |
| Awake without any stimuli (single-digit) |  |  |
| Almost fully conscious but not normal | 1 | 15 |
| Unable to recognize the time, place, and person | 2 | 14 |
| Unable to recall their name or date of birth | 3 | 13 |
| Arousable by some stimuli but reverts to the previous state if the stimulus stops (double-digits) |  |  |
| Arousable by being spoken to | 10 | 12 |
| Arousable by a loud voice | 20 | 12 |
| Arousable only by repeated mechanical stimuli | 30 | 9 |
| Unarousable by any forceful stimuli (triple-digits) |  |  |
| Unarousable but responds to avoid the stimuli | 100 | 7 |
| Unarousable but responds with slight movements, including decerebrate or decorticate postures | 200 | 6 |
| Does not respond at all | 300 | 3 |

Adapted from Nakajima et al.^13^

Abbreviations: GCS, Glasgow Coma Scale; JCS, Japan Coma Scale.

| **Table S2. Results of the subgroup analyses by OHCA in the weighted cohort** | | | | | | | | | |
| --- | --- | --- | --- | --- | --- | --- | --- | --- | --- |
|  | With OHCA |  |  |  |  | Without OHCA | |  |  |
|  | Thrombolysis | Control | Risk differences | |  | Thrombolysis | Control | Risk differences | |
| Outcomes | (*n* = 247) | (*n* = 444) | (95% CI) | P-value |  | (*n* = 186) | (*n* = 343) | (95% CI) | P-value |
| Primary outcome |  |  |  |  |  |  |  |  |  |
| In-hospital mortality, *n* (%) | 152 (62) | 280 (63) | -1.6 (-9.9 to 6.8) | 0.71 |  | 87 (47) | 179 (52) | -5.2 (-14.2 to 3.9) | 0.26 |
| Secondary outcomes |  |  |  |  |  |  |  |  |  |
| Favorable neurological outcomes, *n* (%) | 82 (33) | 129 (29) | 4.3 (-3.8 to 12.4) | 0.30 |  | 88 (47) | 149 (43) | 3.8 (-5.2 to 12.8) | 0.41 |
| Length of hospital stay, days, mean (SD) | 25 (44) | 23 (33) | 2.3 (-4.1 to 8.7) | 0.49 |  | 31 (82) | 25 (32) | 6.1 (-4.7 to 16.9) | 0.27 |
| Length of VA-ECMO, days, mean (SD) | 3 (3) | 4 (5) | -0.5 (-1.1 to 0.1) | 0.12 |  | 3 (3) | 3 (3) | 0.01 (-0.6 to 0.6) | 0.97 |
| Total hospitalization cost, ×10^3^ dollars, mean (SD) | 29 (25) | 27 (21) | 2.5 (-1.6 to 6.6) | 0.23 |  | 31 (26) | 27 (21) | 3.4 (-0.9 to 7.7) | 0.12 |
| Major bleeding in a critical area or organ, *n* (%) | 14 (6) | 14 (3) | 2.7 (-1.6 to 7.0) | 0.22 |  | 9 (5) | 8 (2) | 2.5 (-0.9 to 5.9) | 0.15 |
| Intracranial bleeding, *n* (%) | 7 (3) | 4 (1) | 2.0 (-1.5 to 5.5) | 0.26 |  | 3 (2) | 4 (1) | 0.4 (-1.9 to 2.6) | 0.75 |
| Blood transfusions, ml, mean (SD) |  |  |  |  |  |  |  |  |  |
| Red blood cells | 2901 (2764) | 2644 (2813) | 257 (-248 to 762) | 0.32 |  | 2853 (2763) | 2627 (2794) | 226 (-343 to 794) | 0.44 |
| Fresh-frozen plasma | 1642 (2202) | 1532 (2100) | 109 (-300 to 519) | 0.60 |  | 1416 (2679) | 1248 (1892) | 168 (-384 to 719) | 0.55 |
| Platelet concentrate | 292 (671) | 288 (527) | 3.3 (-116 to 122) | 0.96 |  | 245 (453) | 309 (586) | -64 (-167 to 39) | 0.22 |

CI, confidence interval; OHCA, out-of-hospital cardiac arrest; SD, standard deviation; VA-ECMO, venoarterial extracorporeal membrane oxygenation.

| **Table S3. Results of the sensitive analyses after excluding the patients in the control group who received systemic thrombolysis after the third day of initiating VA-ECMO** | | | | |
| --- | --- | --- | --- | --- |
|  | Weighted cohort | |  | |
|  | Thrombolysis | Control | Risk differences | |
| Outcomes | (*n* = 422) | (*n* = 758) | (95% CI) | P-value |
| Primary outcome |  |  |  |  |
| In-hospital mortality, *n* (%) | 237 (56) | 451 (59) | -3.3 (-9.8 to 3.2) | 0.32 |
| Secondary outcomes |  |  |  |  |
| Favorable neurological outcomes, *n* (%) | 163 (39) | 258 (34) | 4.6 (-1.6 to 10.8) | 0.14 |
| Length of hospital stay, days, mean (SD) | 27 (62) | 23 (32) | 4.4 (-1.3 to 10.2) | 0.13 |
| Length of VA-ECMO, days, mean (SD) | 3 (3) | 3 (3) | -0.2 (-0.6 to 0.3) | 0.47 |
| Total hospitalization cost, ×10^3^ dollars, mean (SD) | 30 (26) | 26 (20) | 3.6 (0.5 to 6.6) | 0.02 |
| Major bleeding in a critical area or organ, *n* (%) | 22 (5) | 20 (3) | 2.6 (-0.2 to 5.4) | 0.06 |
| Intracranial bleeding, *n* (%) | 10 (2) | 9 (1) | 1.2 (-1.0 to 3.4) | 0.27 |
| Blood transfusions, ml, mean (SD) |  |  |  |  |
| Red blood cells | 2879 (2759) | 2575 (2750) | 304 (-76 to 684) | 0.12 |
| Fresh-frozen plasma | 1541 (2396) | 1396 (2009) | 145 (-189 to 479) | 0.39 |
| Platelet concentrate | 274 (591) | 293 (542) | -19 (-101 to 64) | 0.65 |

CI, confidence interval; SD, standard deviation; VA-ECMO, venoarterial extracorporeal membrane oxygenation.
